# Antimicrobial resistance and genotyping of *Staphylococcus aureus* obtained from food animals in Sichuan Province, China

**DOI:** 10.1186/s12917-021-02884-z

**Published:** 2021-04-26

**Authors:** Ting Gan, Gang Shu, Hualin Fu, Qigui Yan, Wei Zhang, Huaqiao Tang, Lizi Yin, Ling Zhao, Juchun Lin

**Affiliations:** College of Veterinary Medicine, Sichuan Agricultural University, 611130 Chengdu, Sichuan China

**Keywords:** MRSA, MSSA, Antimicrobial resistance gene, Molecular typing

## Abstract

**Background:**

*Staphylococcus aureus* (*S. aureus*), especially methicillin-resistant *Staphylococcus aureus* (MRSA), is considered a common zoonotic pathogen, causing severe infections. The objective of this study was to investigate the antimicrobial susceptibility, resistance genes and molecular epidemiology among MRSA and methicillin-susceptible *Staphylococcus aureus* (MSSA) isolated from food animals in Sichuan Province, China.

**Methods:**

This study was conducted on 236 *S. aureus* isolates. All isolates were subjected to antimicrobial susceptibility testing by using a standard microbroth dilution method. The Polymerase Chain Reaction (PCR) was performed to identify genes encoding the β-lactams resistance (*blaZ*, *mecA*), macrolides (*ermA*, *ermB*, *ermC*) and aminoglycosides (*aacA-aphD*). The molecular structures and genomic relatedness of MRSA isolates were determined by staphylococcal chromosome cassette *mec* (*SCCmec*) typing and pulsed-field gel electrophoresis (PFGE), respectively.

**Results:**

Among 236 isolates, 24 (10.17 %) were recognized as MRSA. MRSA isolates showed different resistance rates to 11 antimicrobials ranging from 33.33 to 100 %, while for MSSA isolates the rates varied from 8.02 to 91.51 %. Multi-drug resistance phenotype was found in all MRSA isolates. The *ermC* gene encoding macrolides-lincosamides-streptogramin B was the most prevalent gene detected in 87.29 % of the *S. aureus* isolates, followed by *ermB* (83.05 %), *blaZ* (63.98 %), *aacA-aphD* (44.07 %), *ermA* (11.44 %) and *mecA* (11.02 %) genes. The prevalence of resistance genes in MRSA isolates was significantly higher than that of MSSA. Regarding the molecular morphology, *SCCmec* III (12/24, 50 %) was the most common *SCCmec* type. Furthermore, the PFGE typing showed that 24 MRSA were divided into 15 cluster groups (A to O), the major pulsotype J encompassed 25 % of MRSA isolates.

**Conclusions:**

The *S. aureus* isolates from food animals in Sichuan province of China have severe antimicrobials resistance with various resistance genes, especially MRSA isolates. Additionally, the genetic pool of MRSA isolates is diverse and complex, and further investigation is necessary.

**Supplementary Information:**

The online version contains supplementary material available at 10.1186/s12917-021-02884-z.

## Background

*Staphylococcus aureus (S. aureus)* is one of the important potential pathogens that can cause acute and chronic diseases, such as mastitis, endocarditis, sepsis, bacteremia, and toxic shock syndrome [1]. It has been reported that *S. aureus* can cause mastitis in dairy cows, dermatitis and sepsis in pigs, septic arthritis and subdermal abscesses in poultry [2]. Antibiotic therapy plays an important role in controlling infections caused by *S. aureus*. However, excessive use of antibiotics has resulted the development of resistant *S. aureus* strains, especially methicillin-resistant *Staphylococcus aureus* (MRSA), posing severe threat to humans and animals health [3]. Methicillin-susceptible *Staphylococcus aureus* (MSSA) generally evolves to MRSA through the acquisition and insertion of staphylococcal chromosome cassette *mec* elements (*SCCmec*), which can transfer horizontally and carry resistance gene *mecA* that codes penicillin-binding protein (PBP2a) with low affinity for β-lactam antibiotics [4]. The first occurrence of MRSA was reported in 1961, and the prevalence of MRSA increased year by year. The outbreaks of MRSA has been reported in many countries in the 1980s with high morbidity and mortality [5, 6]. In the USA, MRSA infection has been recognized in more than 320,000 cases and caused more than 10,000 deaths in 2017 [7]. Meanwhile, over 50 % of nosocomial *S. aureus* were associated with MRSA in most of the Asian countries [8]. In China, although the methicillin resistance rate had a trend of reduction since 2005, the prevalence of MRSA was still high [9, 10]. According to CHINET surveillance system in 2019, 31.4 % of all clinical isolates had been recognized as MRSA [10].

Apart from human studies, MRSA has also been known to exist in animals for a long time. MRSA colonies and infections have been reported in domestic livestock, companion animals and wildlife [11]. Prolonged misuse and abuse of antibiotics at farms largely contributed to the wide distribution of MRSA among food animals. It has been revealed that more than 40 % of pigs, 20 % of cattle, and 20–90 % of turkey farms have been affected by MRSA in Germany [12, 13], about 23–32 % of pig farmers were colonized with MRSA in swine farms in the Netherlands [14, 15]. In North America, the prevalence of MRSA had been found to be about 20 % [16]. Whereas in China, the prevalence of MRSA in dairy cow, pigs, and chicken ranches were 6.6 %, 49 %, and 2.1-3.5 %, respectively [17–19]. MRSA in food animals may cause not only animal diseases but also a zoonotic issue between animals and humans through direct contact, environmental contamination, and contaminated animal products [20, 21]. Overall, these findings suggested that the resistance and epidemiological studies of MRSA isolated from animals are necessary for both animal and human health.

Sichuan province is one of the largest producers of food animals, and areas for the production and use of animal drugs. However, there are scant studies regarding the prevalence of MRSA in food animals in Sichuan province. The purpose of the study was to evaluate resistance phenotypes and genotypes of MRSA and MSSA isolates among 236 *S. aureus* isolates from livestock and poultry in Sichuan province. Furthermore, the molecular types of MRSA isolates were analyzed by staphylococcal chromosome cassette *mec* (*SCCmec*) typing and pulsed-field gel electrophoresis (PFGE).

## Results

### Antimicrobial susceptibility testing

**Fig. 1:**
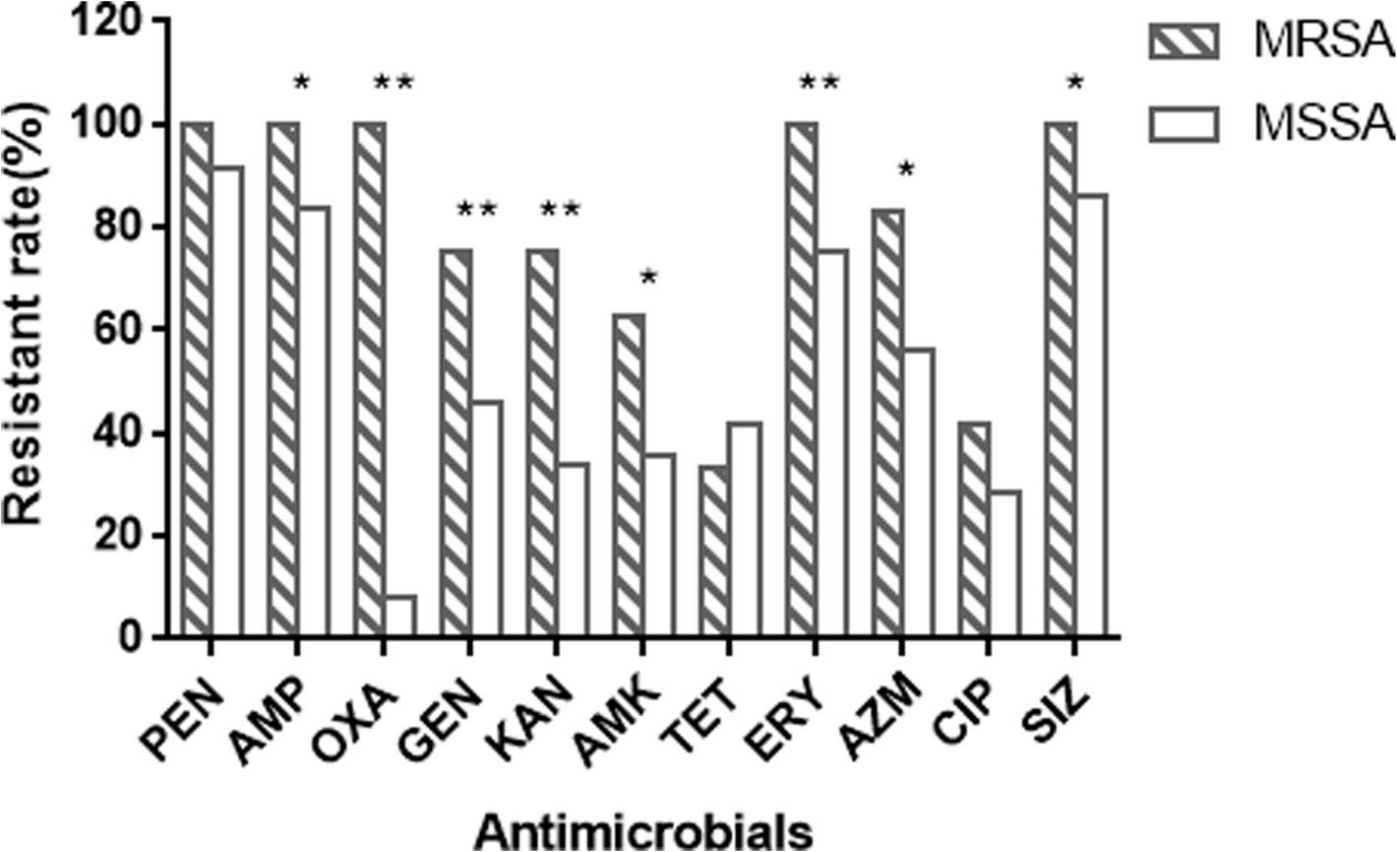
Comparison of resistance of MRSA and MSSA isolates to antimicrobials. PEN, penicillin; AMP, ampicillin; OXA, oxacillin; GEN, gentamicin; KAN, kanamycin; AMK, amikacin; TET, tetracycline; ERY, erythromycin; AZM, azithromycin; CIP, ciprofloxacin; SIZ, sulfafurazole. * indicates significant difference (*P*<0.05), ** indicates extremely significant difference (*P*<0.01) of resistance rate between MRSA and MSSA

**Fig. 2:**
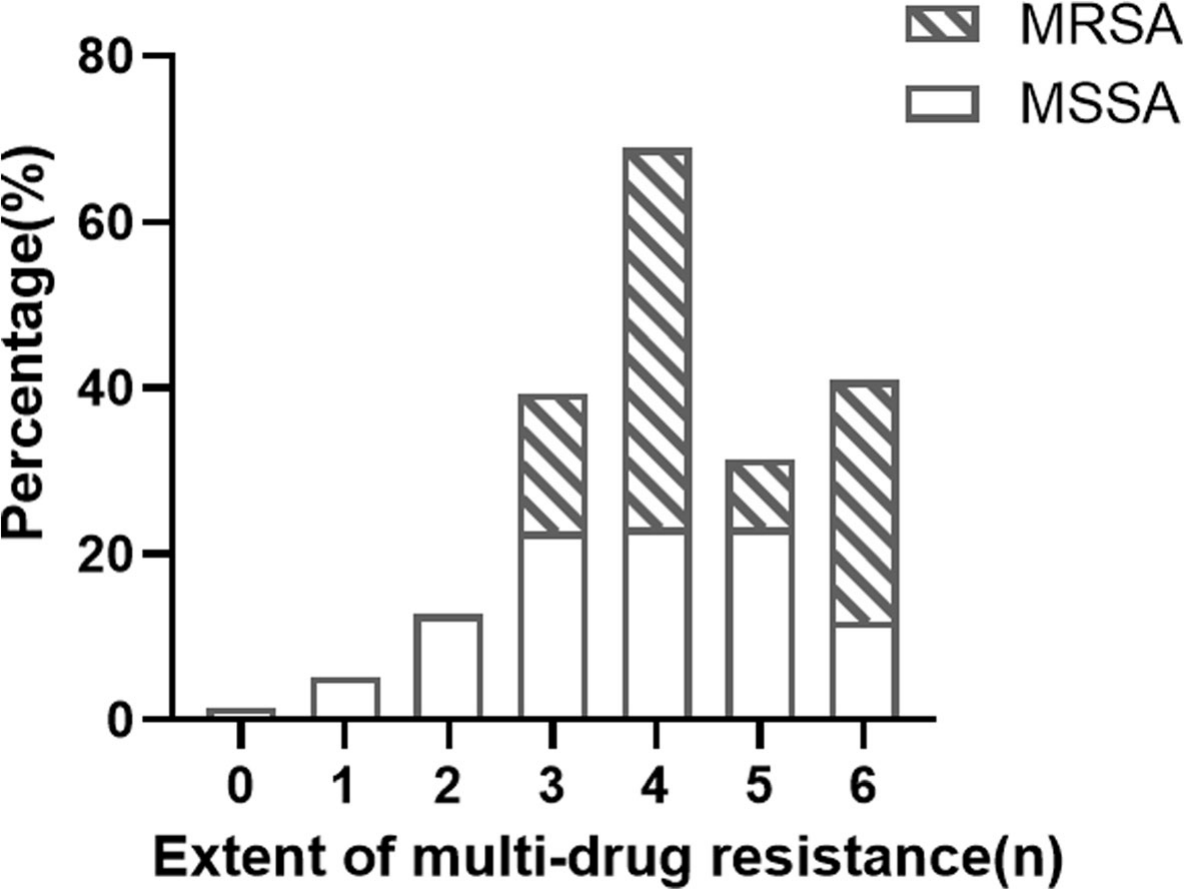
Distribution of multi-drug resistance in MRSA and MSSA isolates. MRSA, methicillin-resistant *Staphylococcus aureus*; MSSA, methicillin-susceptible *Staphylococcus aureus*

There were 236 *S. aureus* isolates isolated from sick chickens (*n* = 97), ducks (*n* = 124), swine (*n* = 11) and cows (*n* = 4). 24/236 (10.17 %) were MRSA and 212/236 (89.83 %) were MSSA. MRSA isolates showed different resistance rates to 11 antimicrobials ranging from 33.33 to 100 %. The serious resistance was not only to β-lactam antibiotics, but also to erythromycin and sulfafurazole (100 % resistance rate each). MRSA isolates were significantly resistant to all other antibiotics except for penicillin, tetracycline, and ciprofloxacin (*P* < 0.05 or *P* < 0.01) compared with MSSA isolates (Fig. 1). Furthermore, 100 % of MRSA isolates were multi-drug resistant whereas only 80.66 % of MSSA showed multi-drug resistance (Fig. 2).

### Detection of resistance genes in ***S. aureus*** isolates

A total of 236 *S. aureus* isolates were tested for six antibiotic resistance genes including the β-lactamases *blaZ* gene (*blaZ*), methicillin resistance determinant (*mecA*), erythromycin ribosome methylase genes (*ermA*, *ermB*, *ermC*) and the bifunctional aminoglycoside N-acetyltransferase and aminoglycoside phosphotransferase (*aacA-aphD*) gene (Table 1). Among these genes, the main genotypes were *ermC, ermB* and *blaZ* (detection rate > 60 %). All MRSA isolates harboured *mecA*, *ermB* and *ermC* genes, while *blaZ*, *ermA* and *aacA-aphD* were detected in 87.50 %, 79.17% and 70.83 % of MRSA, respectively. Subsequent statistical analysis showed that prevalence of all resistance genes in MRSA isolates was significantly higher than that of MSSA (*P* < 0.05 or *P* < 0.01). As shown in Table 2, *aacA-aphD*-positive MRSA isolates showed resistance to aminoglycosides (gentamicin, kanamycin, amikacin) suggesting that the phenotype was in accordance with the genotype. In addition, all MRSA isolates harbored four or more resistance genes in this study. The most common multiple resistance gene combination profile was *blaZ/mecA/ermA/ermB/ermC/aacA-aphD* (54.17 %, 13/24) (Table 2)

**Table 1:** Detection of resistance genes among *S. aureus* isolates

Resistance genes	Percentage of positive isolates (%) (n)		
	MRSA (*n* = 24)	MSSA (*n* = 212)	Total (*n* = 236)
*blaZ*	87.50 (21)*	61.32 (130)	63.98 (151)
*mecA*	100.00 (24)**	0.94 (2)	11.02 (26)
*ermA*	79.17 (19)**	3.77 (8)	11.44 (27)
*ermB*	100.00 (24)*	81.13 (172)	83.05 (196)
*ermC*	100.00 (24)*	85.85 (182)	87.29 (206)
*aacA-aphD*	70.83 (17)**	41.04 (87)	44.07 (104)

*MRSA* methicillin-resistant *Staphylococcus aureus, MSSA* methicillin-susceptible *Staphylococcus aureus.* *indicates significant difference (*P* < 0.05), **indicates extremely significant difference (*P* < 0.01) between MRSA and MSSA

**Table 2:** The resistance genes profile and molecular typing of MRSA isolates in the study

Isolates	Source	Resistance profiles	Resistance genes	PFGE groups	*SCCmec* types
XCCow3	Cattle	PEN/AMP/OXA/ERY/AZM/SIZ	*blaZ/mecA/ermA/ermB/ermC*	A	NT
BMD20	Duck	PEN/AMP/OXA/ERY/SIZ	*blaZ/mecA/ermA/ermB/ermC*	E	III
BMD69	Duck	PEN/AMP/OXA/ERY/AZM/SIZ	*blaZ/mecA/ermA/ermB/ermC*	E	III
BMD23	Duck	PEN/AMP/OXA/GEN/KAN/AMK/ERY/AZM/SIZ	*blaZ/mecA/ermA/ermB/ermC/aacA-aphD*	F	III
BMD9	Duck	PEN/AMP/OXA/ERY/AZM/SIZ	*blaZ/mecA/ermA/ermB/ermC*	G	III
BMD68	Duck	PEN/AMP/OXA/GEN/KAN/AMK/ERY/AZM/SIZ	*blaZ/mecA/ermA/ermB/ermC/aacA-aphD*	I	III
BMD17	Duck	PEN/AMP/OXA/GEN/KAN/AMK/ERY/SIZ	*blaZ/mecA/ermA/ermB/ermC/aacA-aphD*	J	III
BMD18	Duck	PEN/AMP/OXA/GEN/KAN/AMK/ERY/AZM/SIZ	*blaZ/mecA/ermA/ermB/ermC/aacA-aphD*	J	III
BMD24	Duck	PEN/AMP/OXA/GEN/KAN/ERY/AZM/SIZ	*blaZ/mecA/ermA/ermB/ermC/aacA-aphD*	J	III
BMD30	Duck	PEN/AMP/OXA/ERY/AZM/SIZ	*blaZ/mecA/ermA/ermB/ermC*	J	III
BMD41	Duck	PEN/AMP/OXA/GEN/KAN/ERY/AZM/SIZ	*blaZ/mecA/ermA/ermB/ermC/aacA-aphD*	J	III
BMD74	Duck	PEN/AMP/OXA/ERY/AZM/CIP/SIZ	*mecA/ermA/ermB/ermC*	J	III
BMD42	Duck	PEN/AMP/OXA/GEN/KAN/AMK/TET/ERY/AZM/CIP/SIZ	*blaZ/mecA/ermA/ermB/ermC/aacA-aphD*	B	III
YAD2	Duck	PEN/AMP/OXA/GEN/KAN/ERY/AZM/SIZ	*blaZ/mecA/ermA/ermB/ermC/aacA-aphD*	O	V
YAD3	Duck	PEN/AMP/OXA/GEN/KAN/AMK/TET/ERY/AZM/CIP/SIZ	*blaZ/mecA/ermA/ermB/ermC/aacA-aphD*	D	V
YAD4	Duck	PEN/AMP/OXA/GEN/KAN/AMK/TET/ERY/AZM/CIP/SIZ	*blaZ/mecA/ermA/ermB/ermC/aacA-aphD*	C	V
GLD51	Duck	PEN/AMP/OXA/GEN/KAN/AMK/ERY/SIZ	*blaZ/mecA/ ermB/ermC/aacA-aphD*	L	I
GLD54	Duck	PEN/AMP/OXA/GEN/KAN/AMK/ERY/AZM/CIP/SIZ	*blaZ/mecA /ermB/ermC*	L	I
GLD59	Duck	PEN/AMP/OXA/GEN/KAN/AMK/ERY/CIP/SIZ	*blaZ/mecA/ ermB/ermC/aacA-aphD*	H	I
GLD83	Duck	PEN/AMP/OXA/GEN/KAN/AMK/TET/ERY/AZM/CIP/SIZ	*mecA /ermB/ermC/aacA-aphD*	H	I
GLD93	Duck	PEN/AMP/OXA/GEN/KAN/AMK/TET/ERY/AZM/CIP/SIZ	*mecA/ ermB/ermC/aacA-aphD*	M	I
YAC1	Chicken	PEN/AMP/OXA/GEN/KAN/AMK/TET/ERY/AZM/CIP/SIZ	*blaZ/mecA/ermA/ermB/ermC/aacA-aphD*	N	IV
YAC2	Chicken	PEN/AMP/OXA/GEN/KAN/AMK/TET/ERY/AZM/CIP/SIZ	*blaZ/mecA/ermA/ermB/ermC/aacA-aphD*	N	IV
YAC4	Chicken	PEN/AMP/OXA/GEN/KAN/AMK/ERY/AZM/SIZ	*blaZ/mecA/ermA/ermB/ermC/aacA-aphD*	K	NT

*PEN* penicillin, *AMP* ampicillin, *OXA* oxacillin, *GEN* gentamicin, *KAN* kanamycin, *AMK* amikacin, *TET* tetracycline, *ERY* erythromycin, *AZM* azithromycin, *CIP* ciprofloxacin, *SIZ* sulfafurazole, *SCCmec* staphylococcal chromosome cassette *mec,* *PFGE* pulsed-field gel electrophoresis, *NT* non-typeable

### Molecular typing of MRSA isolates

**Fig. 3:**
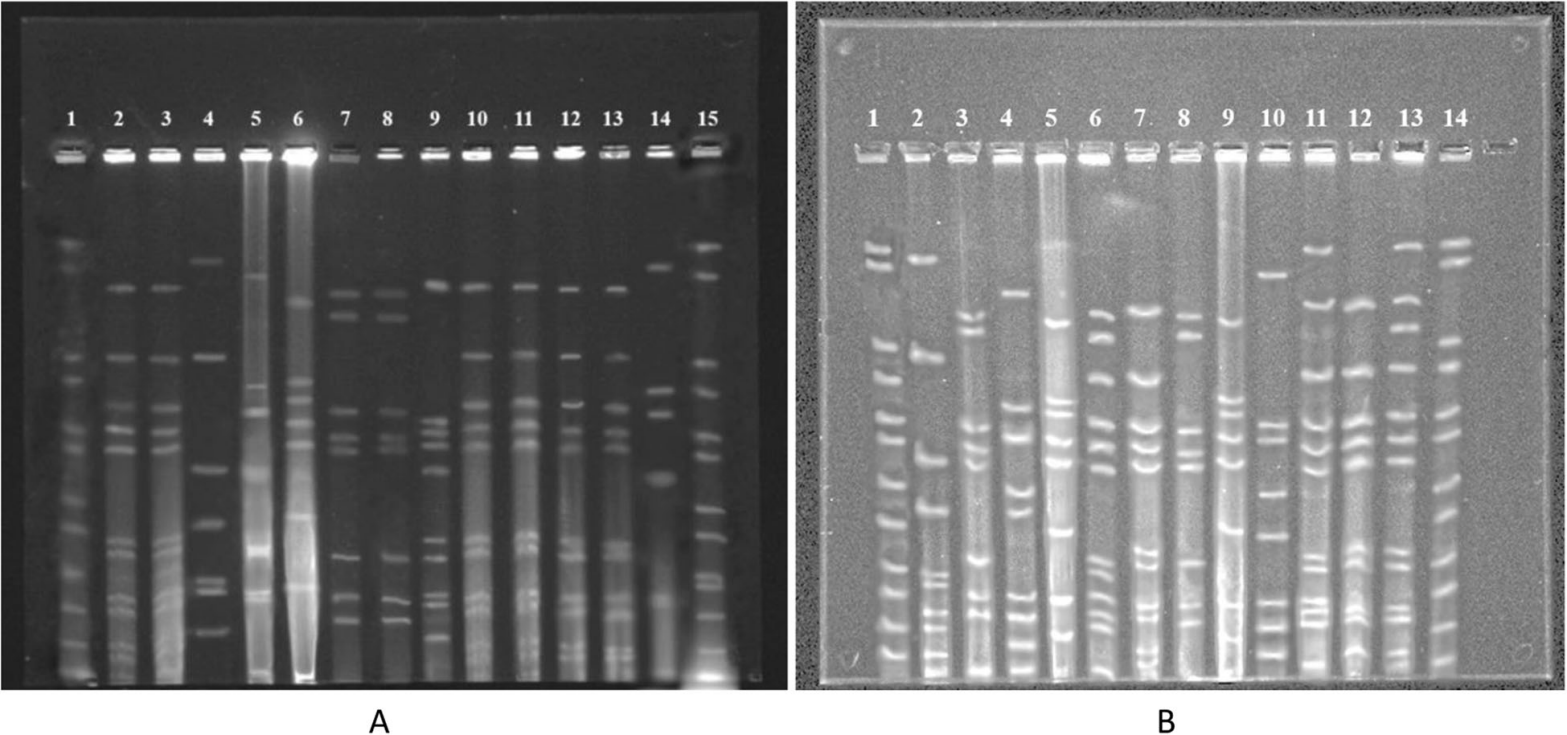
The gels images of MRSA isolates by PFGE typing. **a**: 2, BMD18; 3, BMD24; 4, XCCow3; 5, YAD4; 6, YAD2; 7, GLD51; 8, GLD54; 9, YAC4; 10, BMD41; 11, BMD74; 12, BMD30; 13, BMD17; 14, YAD3; 1 and 15, Xba-digested DNA of *Salmonella* Braenderup H9812 used as DNA molecular size marker; **b**: 2, BMD42; 3, GLD59; 5, YAC1; 6, BMD68; 7, BMD23; 8, GLD83; 9, YAC2; 10, GLD93; 11, BMD9; 12, BMD20; 13, BMD69; 4, error (The forth lane was made by mistake.); 1 and 14, same as the 1 and 15 in **a**

**Fig. 4:**
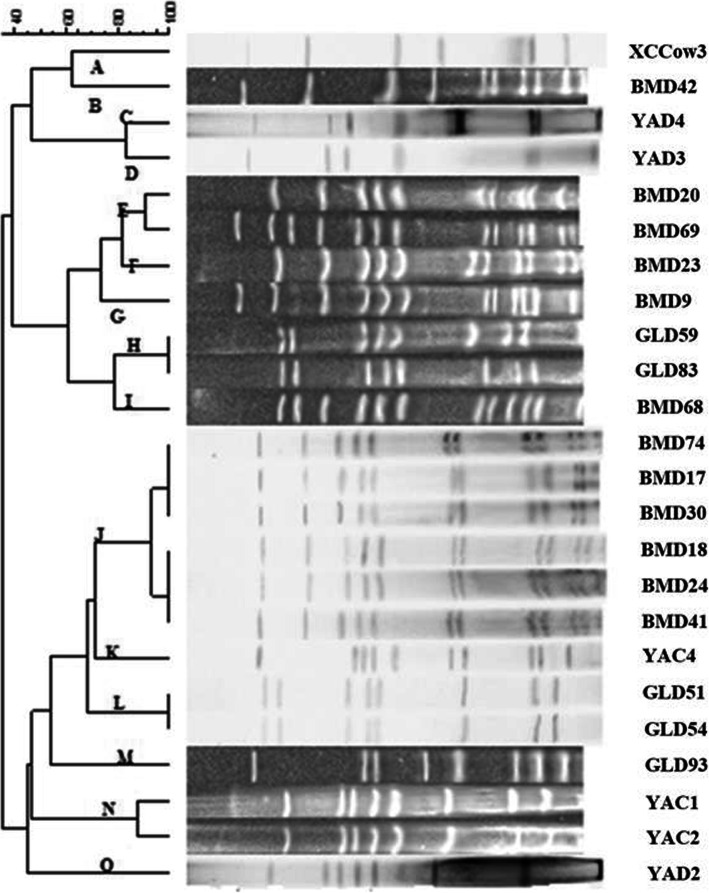
The clusters diagram of 24 MRSA isolates generated by PFGE typing. XCCow3, BMD42, YAD4, YAD3, BMD20, BMD69, BMD23, BMD9, GLD59, GLD83, BMD68, BMD74, BMD17, BMD30, BMD18, BMD24, BMD41, YAC4, GLD51, GLD54, GLD93, YAC1, YAC2, YAD2: strain numbers

The genetic spectrum of the MRSA isolates was varied as revealed by the two typing approaches (Table 2). The characterization of the *SCCmec* cassettes revealed four different types in MRSA isolates: type I, III, IV, and V. *SCCmec* III was identified as the main *SCCmec* type, accounting for 50 % (12/24). Nevertheless, *SCCmec* I, IV, V and unidentified types were detected in 20.83 % (5/24), 8.33 % (2/24), 12.50 % (3/24) and 8.33 % (2/24), respectively. Non-typeable (NT) types were defined as isolates showing unexpected fragments. PFGE of SmaI-digested genomic identified the presence of 15 pulsotypes designated as A to O. PFGE pulsotype J was the largest one, encompassing 25 % (6/24) of isolates, followed by pulsotypes E, H, L and N, each containing two isolates. Each of the remaining pulsotypes (A, B, C, D, F, G, I, K, M, O) contained one isolate (Figs. 3 and 4). As shown in Table 2, the isolates belonging to pulsotype J were all identified as *SCCmec* III, suggesting the results of the two methods were consistent. But compared with *SCCmec* typing, PFGE distinguished the MRSA strains more specifically and detected more types.

## Discussion

Inappropriate empirical use of antibiotics is a major cause of aggravation of drug resistance and poor curative effect. The emergence of MRSA in food animals associates with the presence of MRSA in human consumption foods. Further exploring the mechanisms of resistance and molecular epidemiology of *S. aureus* from animals is a key process in alleviating this crisis. In this study, antimicrobial susceptibility, resistance genes, and molecular epidemiology of the *S. aureus* isolated from food animals in Sichuan Province, China, were characterized.

In order to do a thorough screening of MRSA, we typed each isolate with both phenotype and genotype. The prevalence of MRSA in this study was 10.17 %, which is lower than the 14.2 % in Xinjiang [22], China, the 23.3 % in Brazil [23], and the 13 % in Denmark [24], whereas it is higher than that of the 6.8 % of a previous study in Sichuan [25], the 6.6 % in other provinces of China [17], the 9.2 % in Italy [26], and the 9.8 % in Germany [27]. The difference of prevalence may be due to different regions that were studied or the different species and physical status (healthy or sick) of the animals. Except for oxacillin, *S. aureus* isolates in this study showed high resistance to the antibiotics tested. The high resistance rates imply the misuse of antimicrobials in farms. However, oxacillin and amikacin, which were less used in veterinary medicine, still showed drug resistance, which indicates the potential of cross-resistance. Similar to the findings of Meng et al. [22] and Zayda et al. [28], MRSA isolates were found to be resistant to a broader spectrum of antimicrobials than MSSA isolates in the current study. Resistance rates of the MRSA isolates were significantly higher to gentamicin, kanamycin, amikacin, erythromycin, azithromycin and sulfafurazole compared with those of MSSA isolates, attributing to the existence of *SCCmec* in MRSA strains so that MRSA anchored more resistance genes than MSSA [29, 30]. However, there were no significant differences in the resistance rates of penicillin, tetracycline and ciprofloxacin between MRSA and MSSA (*P* > 0.05). The penicillin resistance rate was extremely high in both MSSA and MRSA isolates. The long-term use of antimicrobials is one of main driving force towards antimicrobial resistance. The reason behind the stable resistance rate maybe because penicillin, being a commonly used drug, was widely preferred for the treatment of Staphylococcal infections for a long time and established considerably stable resistance in *S. aureus*. Hence, the majority of *S. aureus* (either MRSA or MSSA) harbour the penicillinase encoded by *blaZ* that can hydrolyse penicillin. Multi-drug resistance (MDR) was defined as resistance to three or more families of antibiotics. Further comparison of MDR in MRSA and MSSA isolates, MDR was identified in all MRSA isolates and the proportion of multi-drug resistant strains in MRSA was almost three times higher than that of MSSA, which was in line with a previous study [31]. All these findings indicate the severity of antimicrobial resistance of MRSA isolates, which may be attributed to the increase in affinity of MRSA in acquiring mobile genetic elements (MGEs), such as transposons or conjugative plasmids carrying antimicrobial resistance genes [28, 30].

In our study, the majority of isolates possessed 1 to 6 resistance genes. The *ermC* gene was the most common one, which was detected in 87.29 % of *S. aureus* isolates. This finding is consistent with a previous report in China [32], but is different from the another study [33]. The *ermB* and *blaZ* genes were detected in 83.05 and 63.98 % of *S. aureus* isolates, respectively. These results indicate that the majority of *S. aureus* have the ability to acquire the three detected genes. In accord with previous studies [22, 32], a higher proportion of six genes encoding antibiotic-resistance were detected in MRSA isolates than in MSSA isolates. The prevalence of *ermA* and *aacA-aphD* in MRSA was significantly higher than that in MSSA (*P* < 0.01). Nearly 1 % (0.94 %) of isolates harboured the *mecA* gene but showed sensitivity to oxacillin and cefoxitin. This cryptic antibiotic-resistant *S. aureus* has been described in a Taiwanese study involving 91 *S. aureus* isolates with MIC 2.0 µg/ml, 57.1 % of which were *mecA* positive. The study also showed that 3.3 % of 180 *S. aureus* having 1 µg/ml MIC against oxacillin were *mecA* positive isolates [34]. Numerous genes may influence methicillin resistance phenotypes, for example the reason *mecA*-positive *S. aureus* showing sensitivity to oxacillin and cefoxitin may be because β-lactams regulatory genes affect the expression of resistance [35]. Strains with functional *mec* regulatory genes, such as *mecI* and *mecR1* may produce little or no PBP2a, or the expressed protein may be inactivated, leading to a partially or completely suppressed expression of resistance [36, 37]. The *ermB* and *ermC* genes were detected in all MRSA isolates, and the gene *blaZ* was detected in all MRSA except three isolates, while they were only 81.13 %, 85.85% and 61.32 % in MSSA, respectively. Moreover, MRSA carried more abundant multidrug resistance genes compared with those of MSSA. Most of MSSA isolates carried two to four types, while all MRSA carried at least four resistance genes. The broader resistance gene spectrum of MRSA was consistent with its higher drug resistance compared with that of MSSA isolates, suggesting that the phenotype mostly reflects the resistance genotype.

In order to identify the genetic links of strains and to control MRSA infections effectively, it is important to master molecular characteristics. Several molecular typing methods for exploring molecular features of MRSA, such as PFGE, *SCCmec*, the staphylococcal protein A typing (*spa*), multilocus sequence typing (MLST), and so on are available [38–40]. Each of these techniques is used for specific purposes. *SCCmec* is used to recognize the structure and diversity of staphylococcal chromosome cassettes and can be classified as *SCCmec* type I to *SCCmec* XI [41]. MRSA strains may have different accessory genomes and carry different *SCCmec* elements [42]. Traditionally, healthcare-associated MRSA (HA-MRSA) carries *SCCmec* types I–III mainly, while community-associated MRSA (CA-MRSA) or livestock-associated MRSA (LA-MRSA) tend to harbor smaller *SCCmec* elements such as *SCCmec* type IV or type V [43, 44]. In the current study, MRSA isolates from the same areas and source showed the same *SCCmec* type, which may imply that the MRSA genotypes can be different in different regions. Most of the isolated MRSA were found to be *SCCmec* type III (12 isolates, 50 %), in accordance with the conclusion of a previous study that the predominant *SCCmec* type in Asia is type III [29]. However, the isolates in the current study were collected from animals, which may be a case of zoonosis. In addition, we found that the prevalence of combinatorial genotype *blaZ/mecA/ermA/ermB/ermC/aacA-aphD* was significantly higher in *SCCmec* type III, IV, and V, whereas this genotype was not detected in *SCCmec* type I. This finding was in line with the conclusion of a previous study that *SCCmec* type I carried fewer resistance genes [29]. PFGE, including enzyme restriction of bacterial DNA, separation of the restricted DNA bands and clonal assessment of bacteria, is a very sensitive approach for bacterial typing. Fifteen different clusters were obtained by PFGE typing in this study. Overall, no specific relationships had been identified between molecular features and origins. Genetic diversity was noted among animal species and regions, suggesting the complexity of genetic background of the MRSA isolates. All six MRSA isolates of cluster J were attributed to *SCCmec* type III. However, some MRSA isolates, such as YAD2, YAD3 and YAD4, from the same origins and with same resistance genes, were recognized as the same *SCCmec* type, while these were further discriminated into multiple types by PFGE. It has been revealed that PFGE is considered the “gold standard” for bacterial typing [45]. Results of our current study also indicated that the sensitivity of PFGE was higher than that of *SCCmec* typing method.

## Conclusions

These findings revealed that the prevalence of antibiotic resistance of *S. aureus* from food animals is severe in Sichuan province, China, especially the MRSA isolates. MRSA isolates possess a broader spectrum of resistance genes than MSSA does. Additionally, the results of strain characterization suggest that the MRSA isolates from different origins and regions had genetic diversity and complex genetic background. The multiple resistance gene combination of *blaZ/mecA/ermA/ermB/ermC/aacA-aphD* was the most common combination profile in this study. The severity of drug resistance of these *S. aureus* isolates reflects the abuse of antibiotics in food animals. Therefore, it is of great significance to use antibiotics with caution and to strengthen the surveillance of MRSA at farms.

## Methods

### S. aureus isolates and identification of MRSA

**Fig. 5:**
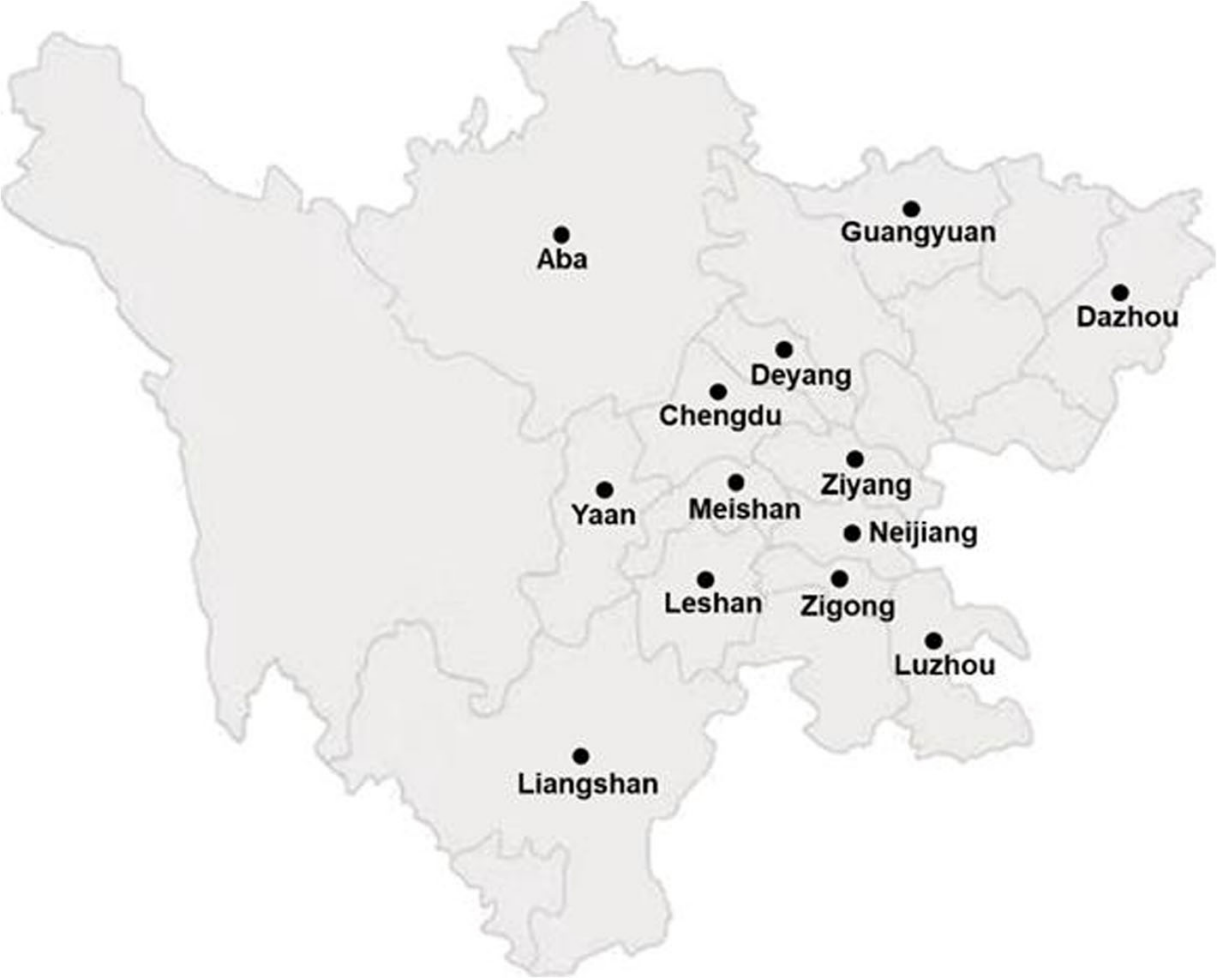
Map of Sichuan showing location of the thirteen areas where *S. aureus*isolates were collected

A total of 236 *S. aureus* isolates were isolated from 13 locations of Sichuan Province, China between 2016 and 2019 (Fig. 5). All isolates were obtained from infected food animals including chickens (*n* = 97 isolates from 1246 samples), ducks (*n* = 124 isolates from 2155 samples), swine (*n* = 11 isolates from 148 samples) and cows (*n* = 4 isolates from 35 samples), sampling from articular exudates, livers, lungs and spleens of chickens and ducks with arthritis, milk of cows with mastitis, and skin swabs of pigs with skin infections and stored in an ice box after sampling for transportation. Samples were incubated at 37℃ in broth containing 1 % tryptone, 7.5 % sodium chloride, 1 % mannitol, and 0.25 % yeast extract for 22–24 h. *S. aureus* recognition was based on the growth status on Mannitol salt agar and CHROMagar™ Staph aureus medium, Gram-staining and standard biochemical tests. Only one isolate per animal sample was chosen for further analysis. The presence of MRSA isolates was confirmed by phenotypic identification methods screening for oxacillin and cefoxitin resistance [46], followed by polymerase chain reaction for detection of *mecA* [47]. The isolates were stored in -80 °C freezer until analysis.

### Antimicrobial susceptibility testing of MRSA and MSSA

Antimicrobial MICs for MRSA and MSSA isolates were determined by broth microdilution and interpreted according to the CLSI guideline [46]. The antimicrobial agents included: penicillin G (PEN), ampicillin (AMP), oxacillin (OXA), gentamicin (GEN), kanamycin (KAN), amikacin (AMK), tetracycline (TET), erythromycin (ERY), azithromycin (AZM), ciprofloxacin (CIP) and sulfafurazole (SIZ). *Escherichia coli* ATCC 25922 and *Staphylococcus aureus* ATCC 25923 (BeNa Culture Collection, Beijing) were used as control strains. Multi-drug resistance (MDR) was defined as resistance to 3 or more families of antibiotics.

### PCR amplification and sequencing of resistance genes of MRSA and MSSA

PCR was used to amplify the β-lactams (*blaZ*, *mecA*), macrolides (*ermA*, *ermB*, *ermC*) and aminoglycosides (*aacA-aphD*) antibiotic resistance genes. Six pairs of primers involved in the PCR reaction (Table 3), the *mecA* primers were cited from a previous study [47], the primers *blaZ*, *ermA*, *ermB*, *ermC* and *aacA-aphD* were designed by software Primer5. E.Z.N.A.™ Bacterial DNA Kit and Plasmid Mini Kit I (OMEGA) were used to extract bacterial DNA according to the manufacturer’s instruction. PCR amplification reactions were conducted in a total volume of 25µL with 1µL of the primer at concentration of 10µmol/L, 12.5µL of 2×Taq PCR MasterMix (TaKaRa, Dalian Co. Ltd), 1µL DNA template, and 9.5µL sterile deionized water. PCR amplification was carried out as follows: 5 min initial denaturation at 95℃, 30 cycles of denaturation at 95℃ for 30 s, annealing for 45 s (see annealing temperature for each gene in Table 3), extension at 72℃ for 45 s and final extension at 72℃ for 15 min. PCR products were analyzed by electrophoresis on 2 % agarose gel containing 0.5 µg/ml of etidium bromide in 0.5X TBE buffer, and the sequencing was determined by a commercial company (Qingke Biotechnology, Chengdu). The DNA sequences were analyzed by the BLAST program, available at the NCBI homepage (http://www.ncbi.nlm.nih.gov/BLAST/).

**Table 3:** The primers used for PCR for resistance genes in *S. aureus* isolates

Primer name	Sequence (5’-3’)	Annealing temperature(℃)	Size (bp)	References
*blaZ -*F	AACACCTGCTGCTTTCGGTA	55.5	314	This study
*blaZ-* R	CACTCTTGGCGGTTTCACTT			
*mecA-*F	CTTTGCTAGAGTAGCACTCG	55.5	533	Herold B.C., et al.
*mecA-*R	GCTAGCCATTCCTTTATCTTG			
*ermA-*F	CTACACTTGGCTTAGGATGA	56.5	311	This study
*ermA-*R	AGTGACTAAAGAAGCGGTAA			
*ermB-*F	TAACGACGAAACTGGCTAA	56.0	414	This study
*ermB-*R	CTGTGGTATGGCGGGTAA			
*ermC-*F	GAGGCTCATAGACGAAGAAA	54.5	375	This study
*ermC-*R	AAGTTCCCAAATTCGAGTAA			
*aacA-aphD-*F	ATTGAAGATTTGCCAGAACA	56.5	178	This study
*aacA-aphD-*R	CACTATCATAACCACTACCG			

*F* forward primer, *R* reverse primer

### Molecular typing of MRSA isolates

*SCCmec* typing for the tested MRSA isolates was determined by the multiplex PCR method described by elsewhere [48]. All MRSA were analyzed by PFGE for their genetic relatedness. PFGE analysis of MRSA isolates tested in the study was as follows: the culture of strains, preparation of agarose gel, DNA digestion by SmaI and electrophoresis, all of which were practiced according to the protocol described by Bannerman et al. [49]. The PFGE banding patterns were interpreted with BioNumerics version 6.0 (Applied Math) by using UPGMA algorithm [50].

### Statistical analysis

Statistical significance for the comparison of resistance rate was determined using X^2^- test by software SAS9.0. *P <* 0.05 was considered to be statistically significant.

## Supplementary Information

**Additional file 1.**

## Data Availability

The DNA sequences were analyzed by the BLAST program, available at the NCBI homepage (http://www.ncbi.nlm.nih.gov/BLAST/).

## Abbreviations

*S. aureus*: *Staphylococcus aureus*
MRSA: Methicillin-resistant *Staphylococcus aureus*
MSSA: Methicillin-susceptible *Staphylococcus aureus*
WHO: World Health Organization
MDR: Multi-drug resistance
MGEs: Mobile genetic elements
PEN: Penicillin G
AMP: Ampicillin
OXA: Oxacillin
GEN: Gentamicin
KAN: Kanamycin
AMK: Amikacin
TET: Tetracycline
ERY: Erythromycin
AZM: Azithromycin
CIP: Ciprofloxacin
SIZ: Sulfafurazole
CLSI: Clinical and Laboratory Standards Institute
MIC: Minimum inhibitory concentration

## References

[1] Wang B, Muir TW (2016). Regulation of virulence in *Staphylococcus aureus*: Molecular mechanisms and remaining puzzles. Cell Chem Biol.

[2] Haag AF, Fitzgerald JR, Penadés JR. *Staphylococcus aureus* in animals. Microbiol Spectr. 2019;7(3):10.1128/microbiolspec.GPP3-0060-2019. 10.1128/microbiolspec.gpp3-0060-2019 PMC11257167 31124433

[3] Petinaki E, Spiliopoulou I (2012). Methicillin-resistant *Staphylococcus aureus* among companion and food-chain animals: impact of human contacts. Clin Microbiol Infect.

[4] Katayama Y, Ito T, Hiramatsu K (2000). A new class of genetic element, staphylococcus cassette chromosome *mec*, encodes methicillin resistance in *Staphylococcus aureus*. Antimicrob Agents Chemother.

[5] Lakhundi S, Zhang K (2018). Methicillin-resistant *Staphylococcus aureus*: Molecular characterization, evolution, and epidemiology. Clin Microbiol Rev.

[6] Mendem SK, Gangadhara TA, Shivannavar CT, Gaddad SM (2016). Antibiotic resistance patterns of *Staphylococcus aureus*: A multi center study from India. Microb Pathog.

[7] CDC. Antibiotic resistance threats in the United States, 2019. Atlanta: US Department of Health and Human Services, CDC. 2019.

[8] Lim WW, Wu P, Bond HS, Wong JY, Ni K, Seto WH (2019). Determinants of methicillin-resistant *Staphylococcus aureus* (MRSA) prevalence in the Asia-Pacific region: A systematic review and meta-analysis. J Glob Antimicrob Resist.

[9] Hu FP, Guo Y, Zhu D, Wang F, Jiang XF, Xu Y (2016). Resistance trends among clinical isolates in China reported from CHINET surveillance of bacterial resistance, 2005–2014. Clin Microbiol Infect.

[10] Hu F, Wang M, Zhu D, Wang F (2020). CHINET efforts to control antibacterial resistance in China. J Glob Antimicrob Resist.

[11] Cuny C, Friedrich AW, Kozytska S, Layer F, Nubel U, Ohlsen K (2010). Emergence of methicillin-resistant *Staphylococcus aureus* (MRSA) in different animal species. Int J Med Microbiol.

[12] Idelevich EA, Lanckohr C, Horn D, Wieler LH, Becker K, Rock R (2016). Multidrug-resistant bacteria in Germany: The impact of sources outside healthcare facilities. Bundesgesundheitsblatt Gesundheitsforschung Gesundheitsschutz.

[13] Köck R, Ballhausen B, Bischoff M, Cuny C, Becker K (2014). The impact of zoonotic MRSA colonization and infection in Germany. Berl Munch Tierarztl Wochenschr.

[14] Lucia vRMM, Van KPH, Kluytmans JA. Increase in a dutch hospital of methicillin-resistant *Staphylococcus aureus* related to animal farming. Clin Infect Dis. 2008;46(2):261–3. 10.1086/524672 18171259

[15] Voss A, Loeffen F, Bakker J, Klaassen CHW, Wulf M (2005). Methicillin-resistant *Staphylococcus aureus* in pig farming. Emerg Infect Dis.

[16] Khanna T, Friendship R, Dewey C, Weese JS (2008). Methicillin resistant *Staphylococcus aureus* colonization in pigs and pig farmers. Vet Microbiol.

[17] Li T, Lu H, Wang X, Gao Q, Dai Y, Shang J (2017). Molecular characteristics of *Staphylococcus aureus* causing bovine mastitis between 2014 and 2015. Front Cell Infect Microbiol..

[18] Sun C, Chen B, Hulth A, Schwarz S, Ji X, Nilsson LE (2019). Genomic analysis of *Staphylococcus aureus* along a pork production chain and in the community, Shandong Province, China. Int J Antimicrob Agents.

[19] Wang X, Tao X, Xia X, Yang B, Xi M, Meng J (2013). *Staphylococcus aureus* and methicillin-resistant *Staphylococcus aureus* in retail raw chicken in China. Food Control.

[20] Aqib AI, Ijaz M, Anjum AA, Malik MAR, Mehmood K, Farooqi SH (2017). Antibiotic susceptibilities and prevalence of Methicillin resistant *Staphylococcus aureus* (MRSA) isolated from bovine milk in Pakistan. Acta Trop.

[21] Erwin V, Jan K (2014). Livestock-associated *Staphylococcus aureus* CC398: Animal reservoirs and human infections. Infect Genet Evol.

[22] Dan M, Yehui W, Qingling M, Jun Q, Xingxing Z, Shuai M (2019). Antimicrobial resistance, virulence gene profile and molecular typing of *Staphylococcus aureus* isolates from dairy cows in Xinjiang Province, northwest China. J Glob Antimicrob Resist.

[23] Guimaraes FF, Manzi MP, Joaquim SF, Richini-Pereira VB, Langoni H (2016). Short communication: Outbreak of methicillin-resistant *Staphylococcus aureus* (MRSA)-associated mastitis in a closed dairy herd. J Dairy Sci.

[24] Tang Y, Larsen J, Kjeldgaard J, Andersen PS, Skov R, Ingmer H (2017). Methicillin-resistant and -susceptible *Staphylococcus aureus* from retail meat in Denmark. Int J Food Microbiol.

[25] Yang X, Liu J, Huang Y, Meng J, Lei G, Jia Y (2018). Prevalence, molecular characterization, and antimicrobial susceptibility of methicillin-resistant *Staphylococcus aureus* from different origins in Sichuan Province, China, 2007–2015. Foodborne Pathog Dis.

[26] Luini M, Cremonesi P, Magro G, Bianchini V, Minozzi G, Castiglioni B (2015). Methicillin-resistant *Staphylococcus aureus* (MRSA) is associated with low within-herd prevalence of intra-mammary infections in dairy cows: Genotyping of isolates. Vet Microbiol.

[27] Monecke S, Ruppelt A, Wendlandt S, Schwarz S, Slickers P, Ehricht R (2013). Genotyping of *Staphylococcus aureus* isolates from diseased poultry. Vet Microbiol.

[28] Zayda MG, Masuda Y, Hammad AM, Honjoh K, Elbagory AM, Miyamoto T (2020). Molecular characterisation of methicillin-resistant (MRSA) and methicillin-susceptible (MSSA) *Staphylococcus aureus* isolated from bovine subclinical mastitis and Egyptian raw milk cheese. Int Dairy J.

[29] Liu J, Chen D, Peters BM, Li L, Li B, Xu Z (2016). Staphylococcal chromosomal cassettes *mec* (*SCCmec*): A mobile genetic element in methicillin-resistant *Staphylococcus aureus*. Microb Pathog.

[30] Deurenberg RH, Vink C, Kalenic S, Friedrich AW, Bruggeman CA, Stobberingh EE (2007). The molecular evolution of methicillin-resistant *Staphylococcus aureus*. Clin Microbiol Infect.

[31] Bernier-Lachance J, Arsenault J, Usongo V, Parent R, Archambault M (2020). Prevalence and characteristics of livestock-associated methicillin-resistant *Staphylococcus aureus* (LA-MRSA) isolated from chicken meat in the province of Quebec, Canada. PLoS ONE.

[32] Wang D, Wang Z, Yan Z, Wu J, Ali T, Li J (2015). Bovine mastitis *Staphylococcus aureus*: Antibiotic susceptibility profile, resistance genes and molecular typing of methicillin-resistant and methicillin-sensitive strains in China. Infect Genet Evol.

[33] Qu Y, Zhao H, Nobrega DB, Cobo ER, Han B, Zhao Z (2019). Molecular epidemiology and distribution of antimicrobial resistance genes of Staphylococcus species isolated from Chinese dairy cows with clinical mastitis. J Dairy Sci.

[34] Chen FJ, Huang IW, Wang CH, Chen PC, Wang HY, Lai JF (2012). *mecA*-Positive *Staphylococcus aureus* with low-level oxacillin MIC in Taiwan. J Clin Microbiol.

[35] Chambers HF (1997). Methicillin resistance in staphylococci: molecular and biochemical basis and clinical implications. Clin Microbiol Rev.

[36] Kuwahara-Arai K, Kondo N, Hori S, Tateda-Suzuki E, Hiramatsu K (1996). Suppression of methicillin resistance in a *mecA*-containing pre-methicillin-resistant *Staphylococcus aureus* strain is caused by the *mecI*-mediated repression of PBP 2’ production. Antimicrob Agents Chemother.

[37] Ryffel C, Kayser FH, Berger-Bachi B (1992). Correlation between regulation of *mecA* transcription and expression of methicillin resistance in staphylococci. Antimicrob Agents Chemother.

[38] Holmes A, Moore LSP, Sundsfjord A, Steinbakk M, Regmi S, Karkey A (2016). Understanding the mechanisms and drivers of antimicrobial resistance. Lancet.

[39] Shopsin B, Gomez M, Montgomery SO, Smith DH, Waddington M, Dodge DE (1999). Evaluation of protein A gene polymorphic region DNA sequencing for typing of *Staphylococcus aureus* strains. J Clin Microbiol.

[40] Enright MC, DN P, Davies CE, Peacock SJ, Spratt BG (2000). Multilocus sequence typing for characterization of methicillin-resistant and methicillin-susceptible clones of *Staphylococcus aureus*. J Clin Microbiol.

[41] Haaber J, Penades JR, Ingmer H (2017). Transfer of antibiotic resistance in *Staphylococcus aureus*. Trends Microbiol.

[42] Florence C, Angeles AM, Wannes V, Katleen H, Freddy H, Patrick B (2013). Transmission dynamics of methicillin-resistant *Staphylococcus aureus* in pigs. Front Microbiol.

[43] Tsai HC, Tao CW, Hsu BM, Yang YY, Chen JS (2020). Multidrug-resistance in methicillin-resistant *Staphylococcus aureus* (MRSA) isolated from a subtropical river contaminated by nearby livestock industries. Ecotoxicol Environ Saf.

[44] Monecke S, Slickers P, Gawlik D, Müller E, Reissig A, Ruppelt-Lorz A (2018). Variability of *SCCmec* elements in livestock-associated CC398 MRSA. Vet Microbiol.

[45] Neoh HM, Tan XE, Sapri HF, Tan TL (2019). Pulsed-field gel electrophoresis (PFGE): A review of the “gold standard” for bacteria typing and current alternatives. Infect Genet Evol.

[46] CLSI. Performance Standards for Antimicrobial Susceptibility Testing. 30th ed. CLSI supplement M100 Wayne: Clinical and Laboratory Standards Institute. 2020.

[47] Herold BC, Immergluck LC, Maranan MC, Lauderdale DS, Gaskin RE, Boylevavra S (1998). Community-acquired methicillin-resistant *Staphylococcus aureus* in children with no identified predisposing risk. JAMA.

[48] Havaei SA, Ghanbari F, Rastegari AA, Azimian A, Khademi F, Hosseini NS (2014). Molecular typing of hospital-acquired *Staphylococcus aureus* isolated from Isfahan, Iran. Int Sch Res Notices.

[49] Bannerman TL, Hancock G, Tenover FC, Miller JM (1995). Pulsed-field gel electrophoresis as a replacement for bacteriophage typing of *Staphylococcus aureus*. J Clin Microbiol.

[50] Wang W, Liu F, Zulqarnain B, Zhang CS, Ke MA, Peng ZX, et al. Genotypic characterization of methicillin-resistant *Staphylococcus aureus* isolated from pigs and retail foods in China. Biomed Environ Sci. 2007;30(8):570–580. 10.3967/bes2017.076 28807097

